# Effects of Interleukin-1β in Glycinergic Transmission at the Central Amygdala

**DOI:** 10.3389/fphar.2021.613105

**Published:** 2021-03-05

**Authors:** Jocelyn Solorza, Carolina A. Oliva, Karen Castillo, Gabriela Amestica, María Constanza Maldifassi, Xaviera A. López-Cortés, Rafael Barra, Jimmy Stehberg, Matthias Piesche, Patricio Sáez-Briones, Wendy González, Mauricio Arenas-Salinas, Trinidad A. Mariqueo

**Affiliations:** ^1^ Center for Medical Research, Laboratory of Neuropharmacology, School of Medicine, Universidad de Talca, Talca, Chile; ^2^ Centro de Bioinformática, Simulación y Modelado (CBSM), Facultad de Ingeniería, Universidad de Talca, Talca, Chile; ^3^ Institute of Biomedical Sciences, Faculty of Medicine, Universidad Andrés Bello, Santiago, Chile; ^4^ Centro Interdisciplinario de Neurociencia de Valparaíso, Facultad de Ciencias, Universidad de Valparaíso, Valparaíso, Chile; ^5^ Department of Computer Science and Industries, Faculty of Engineering Science, Universidad Católica del Maule, Talca, Chile; ^6^ Centro de Investigación Biomédica y Aplicada (CIBAP), Escuela de Medicina, Facultad de Ciencias Médicas, Universidad de Santiago de Chile (USACH), Santiago, Chile; ^7^ Faculty of Biological Sciences and Faculty of Medicine, Instituto de Ciencias Biomédicas, Universidad Andres Bello, Santiago, Chile; ^8^ Laboratory of Biomedical Research, Medicine Faculty, Universidad Católica del Maule, Talca, Chile; ^9^ Oncology Center, Medicine Faculty, Universidad Católica del Maule, Talca, Chile; ^10^ Laboratory of Neuropharmacology and Behavior, School of Medicine, Faculty of Medical Sciences, Universidad de Santiago de Chile (USACH), Santiago, Chile; ^11^ Millennium Nucleus of Ion Channels-Associated Diseases (MiNICAD), Universidad de Talca, Talca, Chile

**Keywords:** interleukin-1β, auxiliary subunit, glycine receptors, beta subunit, central amygdala (CeA), neuroimmune communication

## Abstract

Interleukin-1β (IL-1β) is an important cytokine that modulates peripheral and central pain sensitization at the spinal level. Among its effects, it increases spinal cord excitability by reducing inhibitory Glycinergic and GABAergic neurotransmission. In the brain, IL-1β is released by glial cells in regions associated with pain processing during neuropathic pain. It also has important roles in neuroinflammation and in regulating NMDA receptor activity required for learning and memory. The modulation of glycine-mediated inhibitory activity via IL-1β may play a critical role in the perception of different levels of pain. The central nucleus of the amygdala (CeA) participates in receiving and processing pain information. Interestingly, this nucleus is enriched in the regulatory auxiliary glycine receptor (GlyR) β subunit (βGlyR); however, no studies have evaluated the effect of IL-1β on glycinergic neurotransmission in the brain. Hence, we hypothesized that IL-1β may modulate GlyR-mediated inhibitory activity via interactions with the βGlyR subunit. Our results show that the application of IL-1β (10 ng/ml) to CeA brain slices has a biphasic effect; transiently increases and then reduces sIPSC amplitude of CeA glycinergic currents. Additionally, we performed molecular docking, site-directed mutagenesis, and whole-cell voltage-clamp electrophysiological experiments in HEK cells transfected with GlyRs containing different GlyR subunits. These data indicate that IL-1β modulates GlyR activity by establishing hydrogen bonds with at least one key amino acid residue located in the back of the loop C at the ECD domain of the βGlyR subunit. The present results suggest that IL-1β in the CeA controls glycinergic neurotransmission, possibly via interactions with the βGlyR subunit. This effect could be relevant for understanding how IL-1β released by glia modulates central processing of pain, learning and memory, and is involved in neuroinflammation.

## Introduction

The Central Nervous System (CNS) performs an orchestrated innate immune response to painful injury (Watkins and Maier, 2002; Xu et al., 2020). During chronic pain development, several inflammatory mediators participate in the loss of spinal and supra-spinal inhibition, leading to hyperexcitability of pain-associated circuits (Ji et al., 2018; Maydych, 2019; Mariqueo and Zúñiga-Hernández, 2020). Proinflammatory cytokines including IL-1β, participate actively in pain sensitization (Ferreira et al., 1988; Sommer and Kress, 2004), inducing hyperalgesia and allodynia (Tadano et al., 1999; Wei et al., 2012). These effects are mediated via spinal modulation of GABAergic and glycinergic inhibitory synaptic transmission (Kawasaki et al., 2008; Chirila et al., 2014; Patrizio et al., 2017), by decreasing glycine receptor activity in lamina II postsynaptic interneurons (Chirila et al., 2014), and hence, reducing glycinergic inhibitory control of spinal excitability.

While the role of spinal IL-1β in pain processing has been widely studied, information on the effects of IL-1β at the supra-spinal level is rather precarious. In the brain, IL-1β is released by astrocytes and microglia in response to various proinflammatory stimuli (Sugama et al., 2011; González et al., 2014). Increased IL-1β in brain regions relevant for pain processing such as the hippocampus, prefrontal cortex and amygdala have been reported during neuropathic pain establishment (del Rey et al., 2011; Al-Amin et al., 2011; Gui et al., 2016).

No study to date has evaluated the effects of IL-1β on glycinergic activity at supra-spinal regions. Together with GABA_A_ receptors, GlyRs regulate neuronal excitability in the CNS, including regions that are critical for central nociceptive processing, such as the spinal cord (Gradwell et al., 2017), thalamus (Molchanova et al., 2018), prefrontal cortex (Liu et al., 2015), nucleus accumbens (Muñoz et al., 2018), hippocampus (Aroeira et al., 2011), periaqueductal gray (PAG) (Choi et al., 2013) and amygdala (Delaney et al., 2010).

Glycine receptors are pentameric ligand-gated chloride channels that control synaptic inhibition in the CNS (Thompson et al., 2010; Burgos et al., 2016). GlyRs can be assembled as homopentamers of four types of α subunits (α(1–4)GlyR subunits), or heteropentamers forming complexes with the auxiliary β subunit (βGlyR), in a proposed 3α2β or 2α3β conformation (Grudzinska et al., 2005). The auxiliary βGlyR subunit is unable to form functional channels without αGlyR subunits (Galaz et al., 2015). However, βGlyR affects the establishment of inhibitory synapses by an intracellular interaction with the scaffolding protein Gephyrin (Maric et al., 2011) and modulates the pharmacological profile of glycinergic currents (Bormann et al., 1993). Both αGlyR and βGlyR subunits share common structural characteristics, including a N-terminal extracellular domain (ECD), four transmembrane domains (TMD), and a large intracellular loop (ICD) between TM3 and TM4 (Lynch, 2004).

The role of spinal GlyRs in chronic pain sensitization has been broadly investigated. Using Prostaglandin E2 (PGE2) as a pro-inflammatory mediator to induce peripheral and central inflammatory pain sensitization, it was reported that PGE2 stimulates the intracellular phosphorylation of α3GlyR subunits to reduce glycinergic inhibitory neurotransmission control (Moraga-Cid et al., 2020). The role of α3GlyR subunits in PGE2-induced pain sensitization is further supported by a study showing that PGE2-induced pain sensitization was reduced in mice lacking α3GlyR subunits (*Glra3−/−* Knockout mice) (Harvey et al., 2004). Interestingly, it has been proposed that the auxiliary βGlyR subunit blocks α3GlyR subunit phosphorylation by steric hindrance, as the βGlyR ICD loop is larger than that of α3GlyR subunits (Acuña et al., 2016). The βGlyR subunit shows widespread expression in the CeA nucleus (Delaney et al., 2010), which is involved in the cognitive and emotional integration of peripheral nociceptive pain (Cai et al., 2018). CeA neural circuits integrate cortical inputs to assign valence to peripheral nociceptive stimuli (Pessoa and Adolphs, 2010), and increased excitability in CeA circuits has been associated to reduced inhibitory control during the establishment of neuropathic pain (Ikeda et al., 2007; Jiang et al., 2014). The role of GlyRs at the CeA level has not been explored to date. Given that IL-1β induces hyperalgesia by reducing glycinergic activity in the spinal cord, here we studied whether IL-1β can modulate GlyR activity in the CeA. Furthermore, we evaluated the possibility that IL-1β modulates GlyR activity via interactions with the βGlyR auxiliary subunit.

## Materials and Methods

### Animals

Male Sprague-Dawley (SD) rats (250–350 g) were obtained from the Animal Facility of University of Chile. Animal care and experimental protocols for this study were approved by the Institutional Animal Use Committee of University of Chile (N°17027-MED-UCH) and followed the guidelines for ethical protocols and animal care established by the National Institute of Health, MD, United States and the International Association for the Study of Pain in conscious animals. Every effort was made to minimize animal suffering.

Animals were bred and housed in controlled laboratory conditions, received standard rat chow diet and water *ad libitum* and were housed on a 12-h light/dark cycle at a constant room temperature of 23°C.

### Homology Modeling and Molecular Simulations

We constructed a three-dimensional (3D) molecular model of the heteropentameric GlyR (2α1:3β) based on the X ray crystal structure of α1GlyR subunits from zebrafish (Du et al., 2015a) (Protein Data Bank ID code: 3JAE; Resolution: 3.9 Å). The software MODELLER version 9.18 was used to build the homology models for all sets of receptors (Šali and Blundell, 1993). The GlyR was oriented towards the z axis and embedded in a membrane composed by POPC (1-palmitoyl-2-oleoyl-sn-glycero-3-phosphocholine) lipids. The system was solvated in a water periodic box (162 × 160 × 123 Å) of TIP3 water molecules and ionized at 0.15 mM NaCl. The initial conformations of each system were subjected to cycles of energy minimization of 10,000 steps and further equilibrated by molecular dynamics of 20 ns with α carbons restrained, in order to relax the conformation of the lateral chains and avoid conformation tension generated during the construction of the model. The calculations were performed using NAMD software and the CHARMM36 united-atom force field for membrane lipids and the CHARMM27 force field with the CMAP correction for proteins (Phillips et al., 2005). All analyses were performed using the VMD software (Humphrey et al., 1996). To study the interaction of IL-1β with the GlyR, the crystal structure of the IL-1β Receptor complex bound to IL-1β (ID code: 3O4O; Resolution: 3.3 Å; RValue: 0.29) was used to build the IL-1β homology model. All models were validated using PROCHEK (Laskowski et al., 1993).

### Docking Simulations

Protein-protein interactions between GlyR with IL-1β were explored. The system was named 2α1:3β-IL-1β (heteropentameric α1βGlyR conformation bound to IL-1β). In order to study the putative interactions between IL-1β and the 2α1:3β GlyR the ClusPro2.0 server was used (https://cluspro.org). ClusPro2.0 is based on three key computational stages. First, an extensive sampling of conformations on a rigid-body docking was performed. Then, resulting conformations were grouped by RMSD according to those with the lowest energy, and finally, a refinement of the structures was performed by energy minimizations. Clusters were classified using electrostatic and Van der Waals interactions. Clusters with sizes greater than the sum of their average plus twice their standard deviation were considered representative, as previously reported (Bottegoni et al., 2006).

### Site Directed Mutagenesis

Point mutations in the βGlyR subunit were made using a QuikChange kit from Agilent Technologies (Santa Clara, CA, United States), following the manufacturer’s instructions, and checked by sequencing (Macrogen), using the following primers GlyRY240A: Forward: tga​tat​taa​aaa​gga​gga​tat​cga​agc​tgg​caa​ctg​tac​aaa​at and Reverse: att​ttg​tac​agt​tgc​cag​ctt​cga​tat​cct​cct​ttt​taa​tat​ca.

### Cell Culture and Expression of Recombinant GlyRs

Human embryonic kidney 293 cells (HEK293T) were cultured in Dulbecco’s modified Eagle’s medium supplemented with L-glutamine DMEM-GlutaMAX (Gibco) and 10% fetal bovine serum (Lonza Basel, Switzerland) at 37 °C and 5% CO_2_. Once the cells reached 60–70% confluence, they were transfected with a pcDNA3.1 plasmid containing either the rat αGlyR subunit, rat βGlyR subunit (NovoPro Biosciences) or site mutated rat Y240AβGlyR subunits, using the Effectene transfection kit (Qiagen). GlyR subunits were co-transfected with CD-8 as reporter, and labeled using Dynabeads coupled with anti-CD8 antibody (Invitrogen). Cells were transfected with 0.3 μg total DNA at a co-transfection ratio 1:2:0.1 or 1:0.1 for heteropentameric α1GlyR:βGly:CD8 or homopentameric α1GlyR:CD8, respectively. Cell cultures were incubated for 24 h with the Effectene-DNA complexes, followed by trypsinization and seeding in poly-l coated 12 mm coverslips at a density of 300,000 cells/µl. Electrophysiology experiments were performed 48 h after transfection, in 12-mm glass coverslips coated with poly-lysine (Sigma-Aldrich).

### Patch Clamp Recordings in Cultured Cells

Patch Clamp recordings in whole cell configuration where used to measure evoked current amplitudes on transiently transfected HEK293T cells at −60 mV holding potential. Patch electrodes (3.0–5.0 MΩ) were pulled from borosilicate glass (World Precision Instruments, INC 1B150F-4) in a Sutter P97 puller (Sutter Instruments, Novato, CA, United States) and filled with an internal solution buffer composed of (in mM): 140 CsCl, 10 BAPTA, 10 HEPES, 4 MgCl_2_, 2 ATP, 0.5 GTP. The external solution buffer was composed of (in mM): 140 NaCl, 3 KCl, 10 HEPES, 1.3 Mg_2_Cl and 2.4 CaCl_2_ and 10 glucose. Axopatch 200B amplifier (Molecular Devices, Sunnyvale, CA, United States) in conjunction with an I/O board NI 6221 PCI (National Instruments) and the Strathclyde electrophysiology software WinWCP (University of Strathclyde, UK) were used for data acquisition. EC_50_ and nHill values were obtained experimentally from a normalized dose response curve using data points from the evoked currents in response to 30, 50, 100, 300 and 1,000 µM of glycine, using a nonlinear algorithm in the GraphPad Prism 6 software. The dose-response curves were fitted to Hill’s equation:

$$Iglycine=Imax*\frac{\left[glycine\right]}{EC_{50}^{nHill}+{\left[glycine\right]}^{nHill}}$$

The current evoked at a determined concentration (I glycine), the maximal current obtained at saturating concentrations of glycine (I max), the concentration of glycine to reach the half maximal current response (EC_50_) and the Hill coefficient (nHill), which indicates cooperativity in the binding of glycine to GlyR (Hussein et al., 2019), were estimated from the dose response curves. Perfusion experiments were performed by evoking currents with 50 µM glycine pulses. Then IL-1β (10 ng/ml) was perfused for 60 s, followed by intermittent 50 µM glycine pulses in 10 s intervals until sensitivity was restored. All experiments were performed at room temperature (20°–21°C). These currents were analyzed by fitting a single exponential function to adjust the decay time constant.

### Patch Clamp Recordings in Central Amygdala Slices: Rat Brain Slice Preparation and Electrophysiological Measurements

Four male SD rats were anesthetized with isoflurane and then decapitated. Their brains were rapidly removed and placed in a beaker with cold artificial cerebrospinal solution (ACSF) composed of (in mM): 85 NaCl, 75 sucrose, 3 KCl, 1.25 NaH_2_PO_4_, 25 NaHCO_3_, 10 dextrose, 3.5 MgSO_4_, 0.5 CaCl_2_, 3 sodium pyruvate, 0.5 sodium L-ascorbate and 3 myo-inositol (305 mOsm, pH 7.4 with 95% O_2_/5% CO_2_). Two coronal slices of 300 µm from each hemisphere were obtained from each animal using a vibratome and left for 1 h at 36°C in the ACSF solution. Then the ACSF solution was replaced with a “recording solution,” composed of (in mM): 126 NaCl, 3.5 KCl, 1.25 NaH_2_PO_4_, 25 NaHCO_3_, 10 dextrose, 1 MgSO_4_, 2 CaCl_2_, 3 sodium pyruvate, 0.5 sodium L-ascorbate and 3 myo-inositol (305 mOsm, pH 7.4 with 95% O_2_/5% CO_2_) at room temperature (22°C). Slices were recorded in a submerged-style chamber solution with recording solution at 30–32°C, under an upright infrared-differential interference contrast (IR-DIC) fluorescence microscope (Eclipse FNI, Nikon) equipped with a 40× water objective. Voltage-clamp whole cell recordings were performed in the soma of visually identified neurons in the CeA. We used a borosilicate glass electrode (World Precision Instruments, Sarasota, FL, United States) pulled on a P-97 Flaming/Brown Micropipette Puller (Sutter Instruments, Novato, CA, United States). The glass pipette, ranging from 3.5 to 4.2 MΩ, was filled with intracellular solution containing the following (in mM): 130 Cs-gluconate, 3.5 CsCl, 4 ATP-Mg, 0.3 GTP-Na, 10 Na-phosphocreatine, 1 EGTA, 10 HEPES and 0.4% Biocytin (286 mOsm, pH 7.4 adjusted with CsOH). After seal formation and successful transition to whole-cell configuration, access resistance usually between 4 and 15 MΩ, was continuously monitored; series resistance was monitored and compensated between 75 and 80%. Glycine-mediated currents were isolated in the presence of synaptic blockers CNQX (10 µM), APV (50 µM), and picrotoxin (50 µM) to block AMPAR, NMDAR and GABA_A_ receptors, respectively. The spontaneous inhibitory postsynaptic currents (sIPSCs) obtained as outward currents were recorded at a holding potential of +20 mV. The remaining sIPSCs obtained after further application of bicuculline (10 µM) was considered glycine-based sIPSC. At the end of the recordings, strychnine was applied to block glycinergic currents and thus confirm that the observed currents were glycinergic. We used PClamp detection events to choose single events of about 3–10 pA that were greater than peak to peak noise, to analyze their amplitude, area, and decay constant. Spontaneous synaptic events were detected over 100 s of continuous recording, and two different times following IL-1β application were analyzed (10 ng/ml, at 5 and 15 min). For these experiments, 16 slices out of 4 animals were recorded, and 13 cells were selected for the calculations (the criterium was the recording with stable input resistance (R_in_) along the experiment). Voltage-clamp signals were acquired using a MultiClamp 700B amplifier (Axon CNS, Molecular Devices LLC), low-pass filtered at 10 kHz, digitally sampled at 30 kHz and recorded through a Digidata-1440A interface (Axon CNS, Molecular Devices) and PClamp 10.3 software.

### Statistical Analysis

The results are expressed as mean ± standard error of the mean (SEM). We used one-way analysis of variance (ANOVA) to determine the effects of IL-1β treatment, followed by a Bonferroni’s *post hoc* test. The threshold for statistical significance was set at *p* < 0.05, with a 95% confidence interval. Statistical analyses were performed using Prism software (GraphPad, United States).

## Results

### IL-1β Modulated Glycinergic Currents in Central Amygdala Slices

**FIGURE 1:**
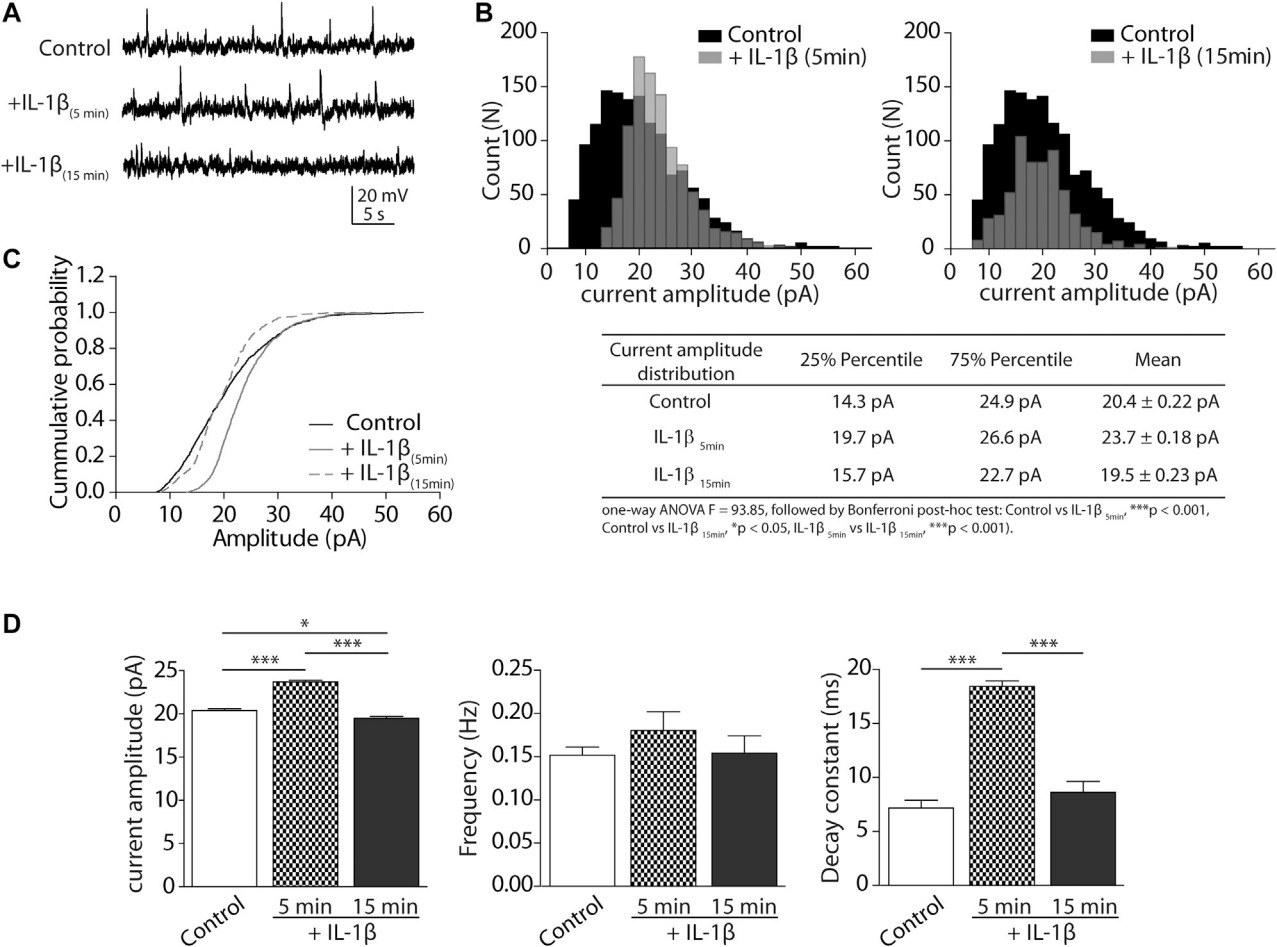
Glycinergic sIPSCs are modulated by IL-1β in CeA neurons of rat. **(A)** Representative synaptic sIPSC glycinergic currents in the presence of CNQX (10 μM), APV (50 μM), picrotoxin (50 µM), bicuculline (10 μM), clamped at +20 mV, 5 and 15 min after incubation with IL-1β. **(B)** Upper: Event distribution histograms of sIPSCs amplitudes before (black bars) and after IL-1β application (gray bars). Below: table with summarized analysis and statistics of distribution histograms **(C)** Cumulative probability histogram of amplitude in CeA neurons before (black line), 5 min (gray line) and 15 min (discontinuous line) after incubation with IL-1β. **(D)** Graphical representation of the mean ± SEM (*n* = 4 rats, 13 cells) of glycinergic sIPSC currents parameters: amplitude (pA), frequency (Hz) and decay time (ms). One-way ANOVA and Bonferroni post-hoc test (*p < 0.05 and ***p < 0.001).

To determine whether IL-1β can modulate GlyR activity in the CeA, we performed whole-cell voltage-clamp recordings in CeA neurons from brain slices. Representative traces of isolated spontaneous inhibitory postsynaptic glycinergic currents (sIPSCs), before (Control) and during the application of IL-1β, are presented in Figure 1A. We generated an amplitude distribution plot of the events recorded in all the slices/cells. We observed that the application of IL-1β induced a significant change in the amplitude distribution of sIPSCs, from the beginning of IL-1β perfusion (5 min), and up to 15 min afterward (Figure 1B). GlyR currents transiently shifted toward events of higher amplitude after 5 min of IL-1β perfusion, and reduced the events in all the amplitudes after 15 min (See the table with summarized values). The cumulative probability distribution of the amplitudes showed that the half-probability is transiently shifted to the right at the beginning of IL-1β perfusion. At 15 min, the half-probability is similar to the control. However, the curve is accelerated by the effects of IL-1β (Figure 1C).

The analysis of the mean current amplitude, frequency, and decay constant (tau) is summarized in Figure 1D. We compared the control average with the 5 and 15 min time points after IL-1β perfusion. The data showed that IL-1β transiently and significantly modulated spontaneous glycine current amplitudes (control: 20.4 ± 0.22 pA; IL-1β 5 min: 23.7 ± 0.18 pA; IL-1β 15 min: 19.5 ± 0.23 pA; *p < 0.05, ***p < 0.001; Figure 1D, left). Instead, IL-1β did not significantly change the frequency of spontaneous glycinergic activity (control: 0.15 ± 0.01 Hz; IL-1β 5 min: 0.18 ± 0.02 Hz; IL-1β 15 min: 0.15 ± 0.02 Hz; Figure 1D, middle). However, the presence of IL-1β did transiently increase the decay time constant of the current (control: 7.2 ± 0.72 ms; IL-1β _5min_: 18.4 ± 0.5 ms; IL-1β _15min_: 8.6 ± 0.9 ms; ***p < 0.001). These analyses suggest that IL-1β can dynamically modulate the amplitude and decay time constant of spontaneous isolated glycinergic currents, suggesting a modulatory effect on GlyR subunits in the CeA.

### The Effects of IL-1β on GlyRs is Mediated by an Interaction with the Auxiliary βGlyR Subunit

**FIGURE 2:**
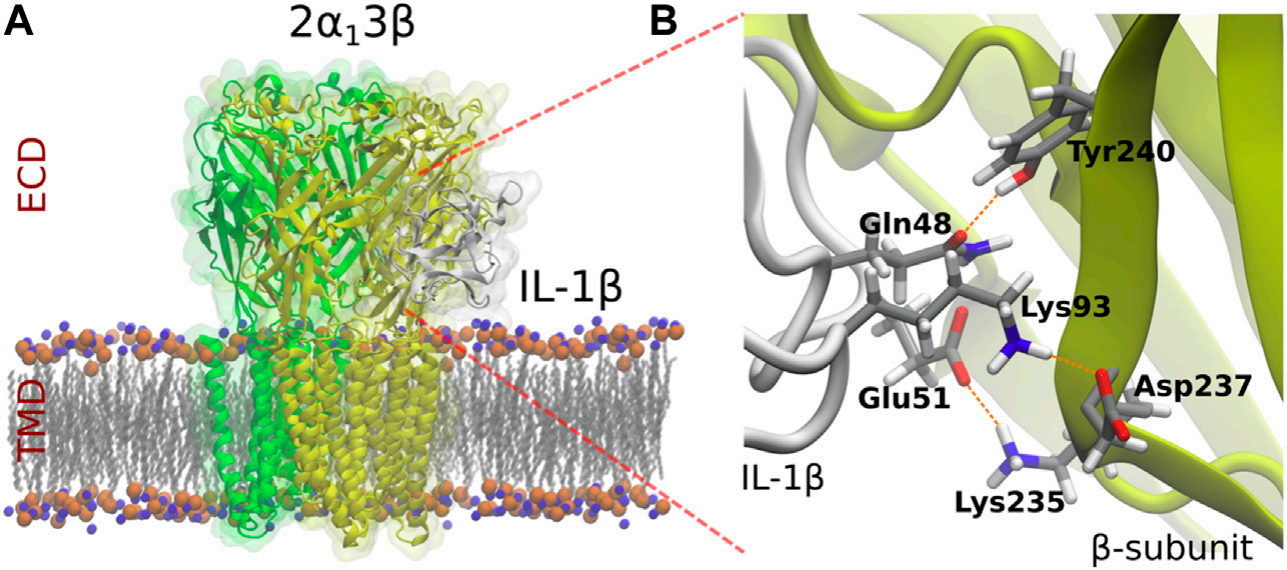
Molecular model of heteropentameric 2α1:3β GlyRs and identification of the residues which putatively interact with IL-1β. αGlyR, βGlyR subunits and IL-1β are represented in green, yellow and grey, respectively.

Previous studies demonstrated that auxiliary βGlyR subunit is broadly expressed in the CeA (Delaney et al., 2010). To determine if βGlyR subunit could be involved in the effects of IL-1β, we constructed a three-dimensional (3D) molecular model of the heteropentameric GlyR (2α1:3β) and IL-1β based on the most complete X-ray crystal structure of α1GlyR available, which was obtained from zebrafish (PDBid: 3JAE) and the IL-1β (PDBid:3o4o). Several three-dimensional reconstructions of αGlyR structure are available, but they are only partial (Du et al., 2015b; Huang et al., 2017; Moraga-Cid et al., 2015). We modeled the ECD and TMD of the βGlyR subunit since there is no available crystallographic structure of the ICD. The sequence identity between both proteins is nearly 50%, which is acceptable for this type of procedure (Burgos et al., 2016). We carried out protein-protein coupling experiments to predict the binding modes of IL-1β on the heteromeric GlyR. The system 2α1:3β-IL-1β showed a representative cluster (*See* Supplementary Appendix S1). Finally, the result was enhanced on Rosie (Rosseta Online Server, https://rosie.graylab.jhu.edu/docking2). The relevant site identified in the interaction with the IL-1β, corresponded to the back of the loop C of β-subunit in the system 2α1:3β. Molecular Docking suggested that the residues Tyrosine 240, Lysine 235 and Aspartate 237 in the back of loop C of the ECD domain of the βGlyR subunit could have a predominant role in the modulation of IL-1β, mostly interacting through hydrogen bonds (Figure 2A). These residues were identified as hot spots for interactions with IL-1β (Wang et al., 2010). Besides, three important putative interactions between β-subunit and IL-1β through hydrogen bonds were described. These interactions correspond to the hydrogen bonds that were established between residues Gln48 (IL-1β)-Tyr240 (βGlyR), Glu51 (IL-1 β)-Lys235 (βGlyR) and Lys93 (IL-1β)-Asp237 (βGlyR), and showed as red dotted lines in Figure 2B. These results suggested that IL-1β may modulate GlyRs currents by interacting with the βGlyR auxiliary subunit, however to prove it we performed site-directed mutations.

### The Modulation of Glycinergic Currents by IL-1β Is Impaired by Site-Directed Mutation in the Auxiliary βGlyR Subunit

**FIGURE 3:**
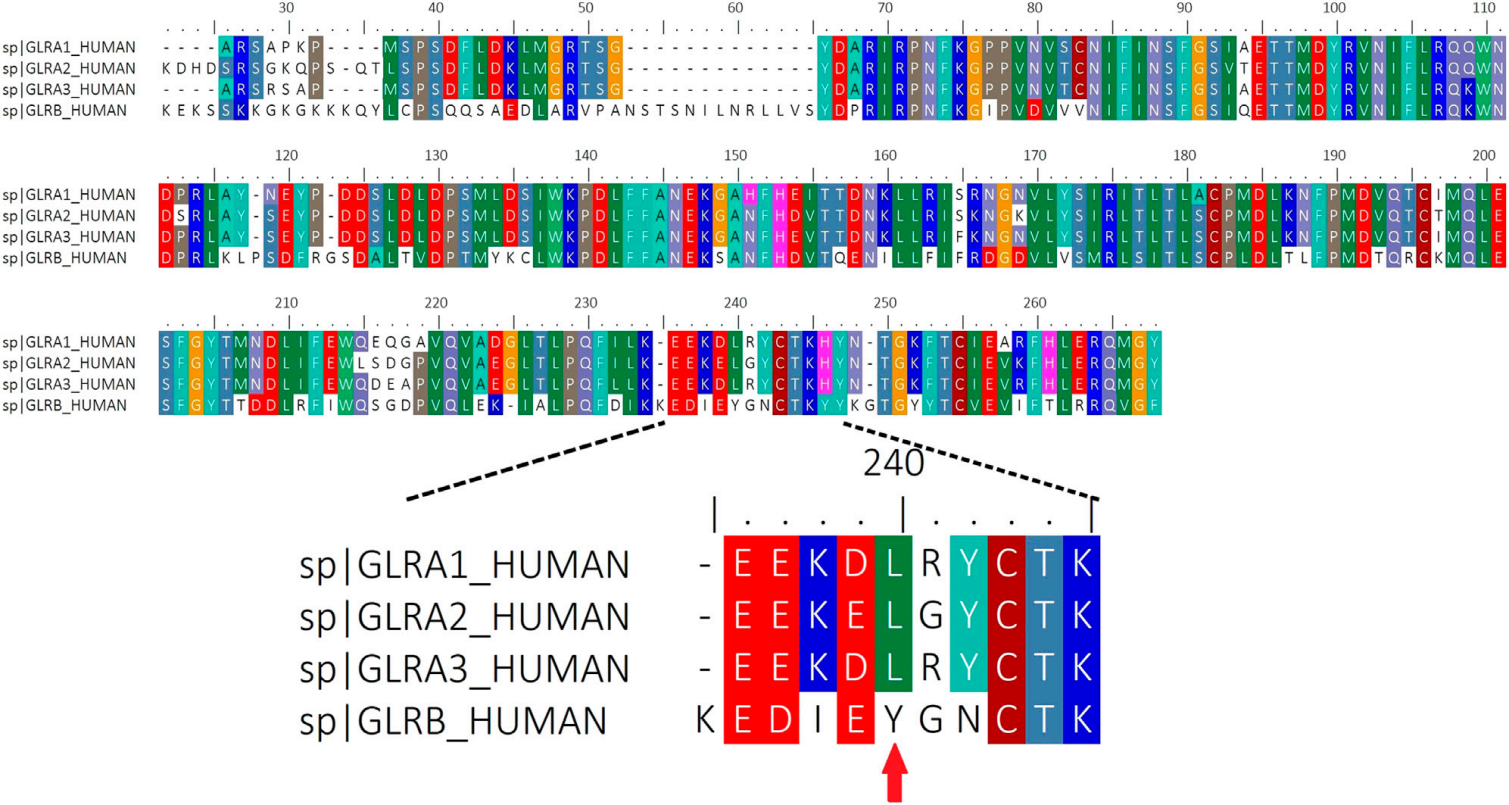
Sequence Alignment of complete Human ECD domains of α(1–3) and βGlyR subunits. Red arrow indicates the location of the residue (Y240) that was mutated in the βGlyR subunit.

**FIGURE 4:**
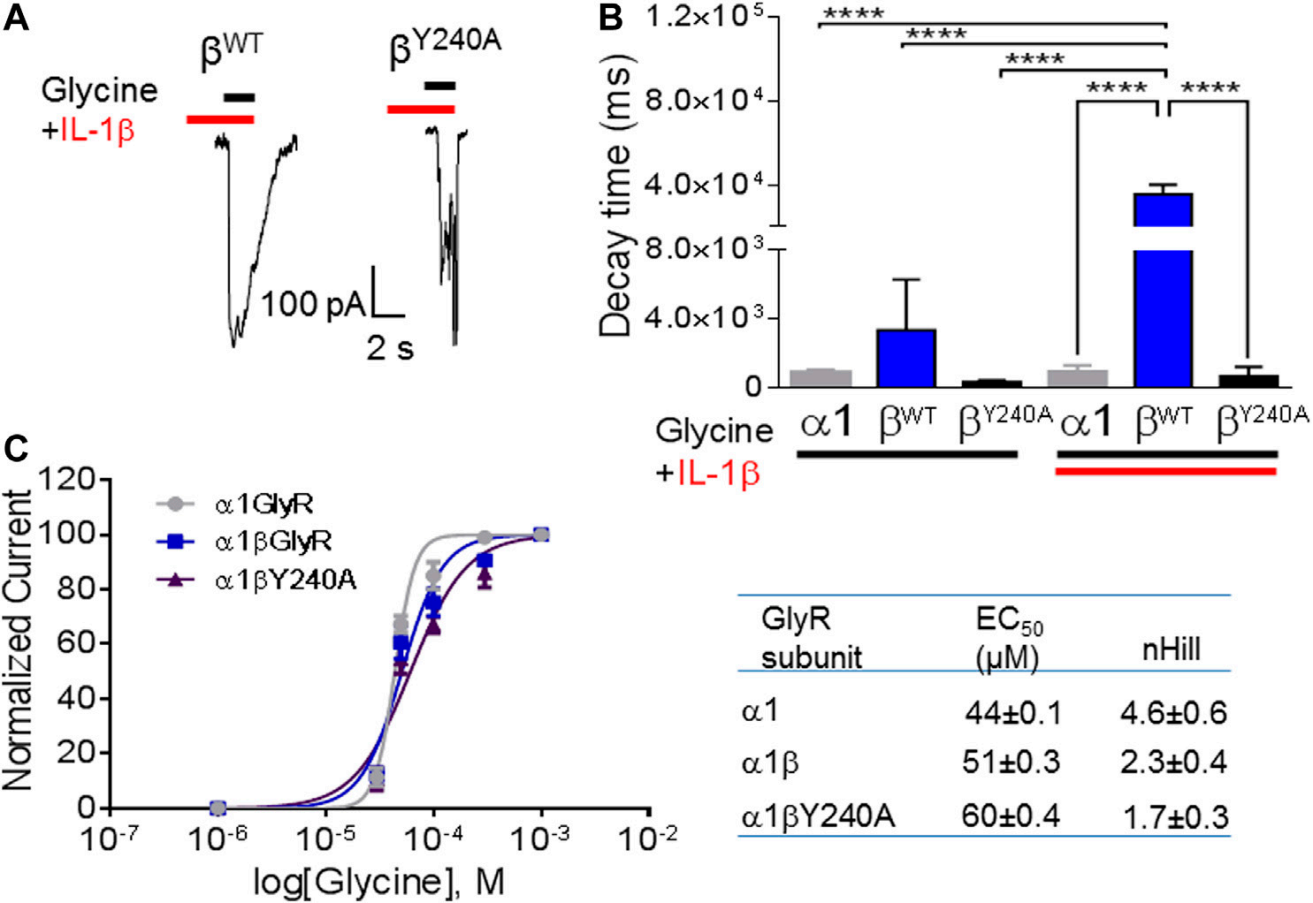
The effects of IL-1β are mediated by interaction with auxiliary βGlyR. **(A)** Representative glycine-activated currents of α1β wild type and α1βY240Α after the perfusion of glycine (black line) + IL-1β (red line). **(B)**: Graphical representation of effects of glycine or glycine + IL-1β in homo and heteropentameric GlyRs. **(C)** Graphical representation of dose-response curve. Glycine activated currents at different glycine concentrations (10–1,000 μM) were normalized to a percentage of the maximal response to the application of glycine 1,000 μM in HEK293T cells transfected with different subunits of GlyRs. Data are presented as mean ± SEM.

We performed an alignment of human ECD domains of αGlyR and βGlyR subunits focusing on the domain that contains the amino acids proposed to interact with IL-1β (Figure 3). The Tyrosine 240 residue that was divergent in βGlyR was selected to perform a site directed mutagenesis. Then, whole-cell patch clamp electrophysiological recordings were performed on HEK293T cells transfected with α1 alone, α1β wild type (α1βWT) or α1β mutated (α1βY240A), and perfused with glycine (50 μM) and IL-1β (10 ng/ml) as already described (Kawasaki et al., 2008) (Figure 4). The heteropentameric α1βWT exhibited greater current decay time constant when compared to the mutated α1βY240A GlyR (Figure 4A; *τ* = 35,420 ± 2,671 ms and *τ* = 3,367 ± 1,684 ms, respectively (*n* = 3 cells from three different cultures; one-way ANOVA, ****p < 0.0001). The plotted current decay time constants of glycine-activated currents in homo- and heteropentameric GlyRs after the application of glycine or glycine + IL-1β are shown in Figure 4B. The effect of IL-1β significantly increases the decay time of the chloride currents in the presence of a βGlyR subunit in heteropentameric GlyRs; whereas, when the βGlyR was mutated (βY240A) the effect of IL-1β was abolished. This data shows that this subunit is important for the cytokine-induced effect in the GlyRs kinetics. Remarkably, it also suggests that the Tyrosine 240 residue from the βGlyR subunit is critical for IL-1β effects on GlyRs (Figure 4B). On the other hand, the EC_50_ for glycine was not affected by the mutated subunit (comparing EC_50_ of α1βWT with α1βY240A; Figure 4C and table), showing that the mutation affects the modulation of GlyRs by IL-1β without altering the pharmacological properties of the channel. Altogether these results suggest that IL-1β modulates GlyR activity through an interaction with the βGlyR subunit.

## Discussion

Here, we showed that IL-1β modulates glycinergic transmission in the CeA, which is in line with previous studies showing that IL-1β decreases spinal glycinergic inhibitory neurotransmission by reducing the number and amplitude of sIPSCs events (Kawasaki et al., 2008; Chirila et al., 2014; Patrizio et al., 2017). We recorded spontaneous currents (sIPSC), this is, spontaneous postsynaptic currents generated in the absence of experimental stimulation, but by action-potential-dependent and the spontaneous release of neurotransmitter. Changes in current frequency are related to vesicular release from presynaptic sites, whereas changes in current amplitude are dependent upon activation of the postsynaptic receptors. Our results show a transient effect of IL-1β in CeA neurons lasting for minutes, suggesting that the modulation of glycinergic transmission induced by IL-1β could be modulatory and dynamic, affecting channel kinetic parameters at postsynaptic sites. Thus, in our analysis, we observed a transient increase in the amplitude of sIPSCs, probably due to increased activation of postsynaptic GlyRs, with a later reduction (after 15 min) due to a reduced activation, or inhibition, of postsynaptic GlyR. No changes related to frequency were observed, suggesting that presynaptic release of glycine was not affected by IL-1β.

Previous evidence demonstrated that clustering of GlyR is dynamically regulated depending on the subunit composition, and its mobility along the synapse is strongly dependent on the presence of α1 and β subunits (Patrizio et al., 2017). Moreover, in that report, the authors showed that IL-1β affects GlyR currents when α1β, but no other α subunits (α2,3) are present, suggesting that the effect of IL-1β is dependent on subunit-specific tissue expression. In the amygdala, the presence of α1GlyR and βGlyR is relevant, specifically in CeA, where the expression of βGlyR subunit is particularly high (Delaney et al., 2010). Our data indicate that in this region, GlyR currents are dynamically modulated by IL-1β, and this effect is specifically dependent on the presence of βGlyR. The amplitude distribution histograms were built two times after IL-1β, where we observed a shift toward the higher events. We suggest that this is part of the dynamic effect of IL-1β in the particular tissue as the amygdala. The unusual high expression of βGlyR subunit in the CeA induces the clustering of GlyRs, transiently increasing the postsynaptic currents. Later, the inhibition caused by IL-1β exerts its effect reducing significantly the currents, disaggregating the clusters. The clustering of GlyRs found in synapses is a process regulated by the auxiliary βGlyR subunit via an intracellular interaction with the scaffolding protein Gephyrin (Maric et al., 2011). The exact mechanism by which IL-1β modulates the GlyRs is unknown; however, we propose that IL-1β exerts a conformational change at the extracellular protein domain that causes changes both in channel opening and receptor clustering. Based on previous studies and our present results, it is possible to propose that IL-1β reduces glycinergic inhibitory control in CeA neurons by disaggregation of glycinergic clusters.

We performed *in silico* docking experiments using the α1βGlyR 3D structure because α1β is the predominant GlyR conformation in the mature central nervous system (Aguayo et al., 2004). Our molecular docking results show that IL-1β may interact with the loop C at the ECD of βGlyR subunits. This structure has been implicated in the conformational rearrangement necessary for the closing of the channel (Huang et al., 2017). Accordingly, our data in transfected HEK cells show that application of IL-1β slows down the recovery of glycine activated currents in the α1βWT. A similar effect was observed in CeA slices, a region with elevated levels of βGlyR, where the decay constant was significantly increased in response to IL-1β. The effect of IL-1β on the glycine current recovery was abolished in channels formed by the βGlyR containing the Y240A mutation. This result supports the idea that IL-1β is interacting with the GlyR in a β subunit dependent binding.

Given that the release of IL-1β is increased in brain regions associated to pain processing including the amygdala in different models of chronic pain (del Rey et al., 2011; Al-Amin et al., 2011; Gui et al., 2016), it is probable that the modulation of glycinergic currents induced by IL-1β may be relevant for pain processing in the CeA. Further studies will be required to assess the role of inhibitory glycinergic currents in the processing of pain at the CeA.

However, the relevance of the present report is not limited to pain processing. The expression of IL-1β has also been reported to increase after LTP induction in the hippocampus (Schneider et al., 1998); while its blockade has been shown to facilitate hippocampus-dependent memory (Yirmiya, 2002; Avital et al., 2003; Depino et al., 2004). The expression of IL-1β in brain regions associated to pain processing, mood, and memory (such as the hippocampus and amygdala) can be associated with chronic pain perception, but also with learning deficits and depression triggered by chronic pain. How the modulation of glycinergic currents by IL-1β in the CeA and other brain regions contributes to learning impairments and mood disorders is a matter of further investigation.

In conclusion, GlyRs activity is modulated by IL-1β in the CeA, possibly via interactions with the βGlyRs auxiliary subunit. This subunit may play a critical role in central pain sensitization. Elucidation of the binding mode between IL-1β and the βGlyR subunit may offer novel possibilities for the development of new pharmacological agents with potential analgesic activity to treat chronic pain and its associated pathologies.

## Highlights

1 IL-1β modulates inhibitory synaptic transmission in the Central Amygdala
2 The effect of IL-1β is mediated by the auxiliary βGlyR subunit

## Data Availability

The raw data supporting the conclusions of this article will be made available by the authors, without undue reservation.
