# Supplementary material for: Effects of Interleukin-1β in Glycinergic Transmission at the Central Amygdala
Source: Front Pharmacol. 2021 Mar 5;12:613105. doi: 10.3389/fphar.2021.613105 (PMC7973117; doi:10.3389/fphar.2021.613105)
Supplement: Supplementary file 1 [file datasheet1.pdf]

Appendix 1

Table 1. Summary results from ClusPro2.0

Cluster	Members	Weighted Score (kcal/mol)
0	128	-199,1
1	191	-213,5
2	110	-209,8
3	21	-215,6
4	37	-225,8
5	22	-224,2
6	22	-207
7	21	-196,4
8	20	-211,5
9	12	-206,5
10	12	-203,8
11	10	-208,6
12	10	-203,2
13	10	-196,1
